# 1432. Patient-Reported Urinary Tract Infection Symptoms Among Veterans with Neurogenic Bladder

**DOI:** 10.1093/ofid/ofab466.1624

**Published:** 2021-12-04

**Authors:** Margaret A Fitzpatrick, Marissa Wirth, Katie J Suda, Stephen Burns, Frances Weaver, Eileen Collins, Nasia Safdar, Rebecca Kartje, Charlesnika T Evans

**Affiliations:** 1 Center of Innovation for Complex Chronic Healthcare, Edward Hines Jr. VA Hospital, Hines, Illinois; 2 Edward Hines Jr. VA Hospital, Hines, Illinois; 3 University of Pittsburgh, Pittsburgh, PA; 4 VA Puget Sound Health Care System, Seattle, Washington; 5 Center of Innovation for Complex Chronic Healthcare, Hines, Illinois; 6 University of Illinois Chicago, Chicago, Illinois; 7 University of Wisconsin-Madison School of Medicine and Public Health, Madison, Wisconsin; 8 HInes VA Hospital, Hines, Illinois; 9 Northwestern University and VA, Hines, Illinois

## Abstract

**Background:**

Urinary tract infections (UTIs) and asymptomatic bacteria (AB) are common in patients with neurogenic bladder (NB) but differentiating between the two is challenging because laboratory tests cannot distinguish AB from UTI. This diagnostic uncertainty can lead to antibiotic overuse. Characterization of patient-reported symptoms from large cohorts of patients with NB can inform interventions to improve appropriate UTI diagnosis and management.

**Methods:**

Retrospective cohort study of 1,797 adults with NB due to spinal cord injury/disorder (SCI/D), multiple sclerosis (MS), and/or Parkinson’s Disease (PD) accounted for 568 patients with UTI encounters (via ICD10) at 4 Veterans Affairs (VA) medical centers between 2017-2018. Demographic and clinical data were collected from national VA datasets. Medical record review was performed on a random sample of 198 encounters. Chi-square/Fisher’s exact test were used to compare symptoms by patient and encounter characteristics.

**Results:**

Among the 198 encounters (mean age=65 years), 33% of patients had SCI/D, 29% PD, 20% MS, and 17% had more than one diagnosis. Most encounters were for men (88%) in inpatient or long-term care settings (62%). 76% of patients used bladder catheters; most indwelling (n=92). Fever was the most frequent symptom (30%), followed by change in urine odor, color, and/or consistency (26%) and lethargy/malaise (21%). Only 38% of encounters had a urinary tract-specific symptom recorded (e.g., dysuria); 81% had non-specific symptoms (e.g., fever, lethargy). 64% of encounters were deemed an appropriate UTI diagnosis. Characteristics in red in Figure 1 were significantly associated with non-specific symptoms (p< 0.05).

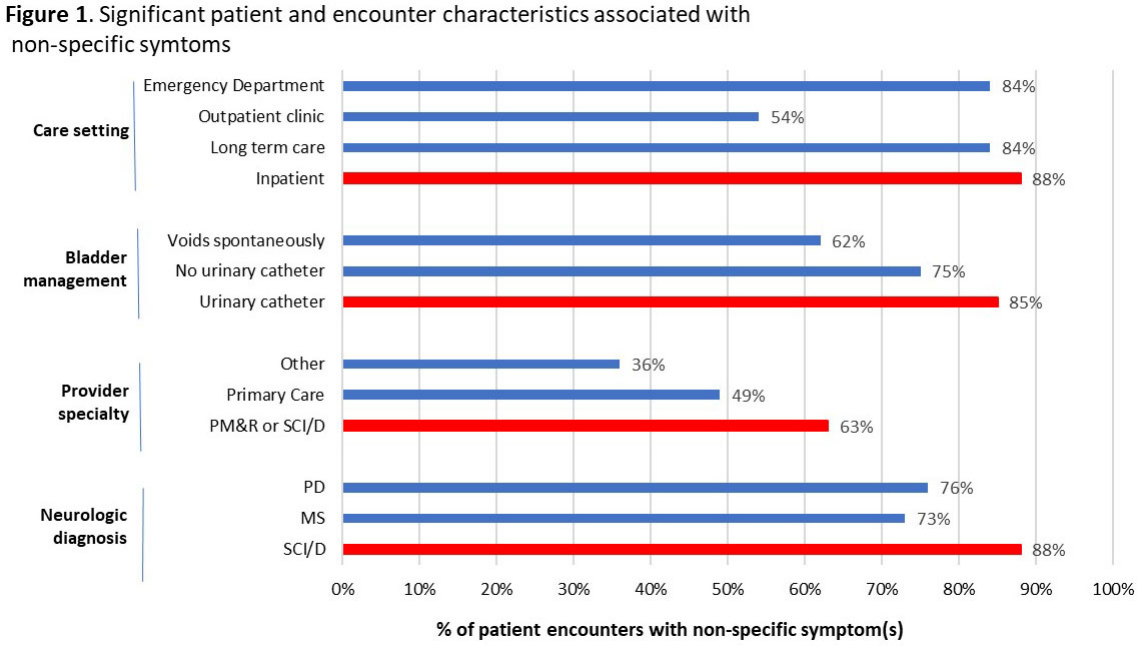

Patient and encounter characteristics found to be significantly associated with non-specific symptoms, p < 0.05.

**Conclusion:**

Symptoms not specific to the urinary tract are the most frequently reported symptoms in patients with NB and encounters with a UTI diagnosis. Change in urine odor/color were reported often; however, guidelines recommend against using these for UTI diagnosis. Providers should ensure that alternate sources of non-specific symptoms are evaluated prior to attributing them to UTI. Antibiotic stewardship interventions targeted to physical medicine and rehabilitation (PM&R) and primary care providers in inpatient settings may improve UTI diagnosis in patients with NB.

**Disclosures:**

**Charlesnika T. Evans, PhD, MPH**, **BioK+** (Consultant)

